# Association between precocious puberty and obesity risk in children: a systematic review and meta-analysis

**DOI:** 10.3389/fped.2023.1226933

**Published:** 2023-08-11

**Authors:** Yongfu Song, Yibu Kong, Xiaofei Xie, Yongji Wang, Na Wang

**Affiliations:** Department of Pediatrics, Hospital Affiliated to Changchun Traditional Chinese Medicine University, Changchun, China

**Keywords:** precocious puberty, general obesity, central obesity, overweight, meta-analysis

## Abstract

**Objectives:**

The aim of this study was to evaluate the potential association between early onset puberty and the risk of different forms of obesity in children.

**Methods:**

The databases PubMed, EMBASE, Web of Science and Cochrane Library were systematically searched for relevant studies. The odds ratio (OR) and 95% confidence interval (CI) of obesity in precocious puberty were calculated using Stata software 14.0. A fixed-effects model was used if *P* > 0.1 and *I*^2^ ≤ 50%. Otherwise, a random-effects model was used. Publication bias was assessed using funnel plots and Egger's test.

**Result:**

The pooling analysis showed that precocious puberty in girls was associated with a higher risk of obesity (OR = 1.98; 95% CI: 1.76–2.24; *I*^2^ = 0.00%, *P *< 0.001). Girls with a history of precocious puberty were found to have an increased risk of general obesity (OR = 2.03; 95% CI: 1.62–2.55; *I*^2^ = 22.2%, *P *< 0.001), central obesity (OR = 1.96; 95% CI: 1.70–2.26; *I*^2 ^= 0.00%, *P *< 0.001), and overweight (OR = 2.03; 95% CI: 1.68–2.46; *I*^2 ^= 5.1%, *P *< 0.001). The pooled analysis showed that precocious puberty in boys was not associated with an increased risk of obesity (OR = 1.14; 95% CI: 0.86–1.51; *I*^2 ^= 50.6%, *P* = 0.369). In boys, the occurrence of precocious puberty was not associated with an elevated risk of general obesity (OR = 0.96; 95% CI: 0.40–2.27; *I*^2^ = 79.6%, *P *= 0.922), central obesity (OR = 1.17; 95% CI: 0.96–1.43; *I*^2 ^= 0.00%, *P *= 0.125), or overweight (OR = 1.03; 95% CI: 0.56–1.88; *I*^2^ = 74.4%, *P *= 0.930).

**Conclusion:**

This meta-analysis suggests that the onset of puberty at an early age in girls is associated with an increased risk of obesity, however precocious puberty in boy was not associated with an increased risk of obesity. These findings highlight that precocious puberty should be considered an independent risk factor for obesity in girls.

**Systematic Review Registration:**

CRD42023404479.

## 1. Introduction

Childhood obesity is a complex public health crisis that is prevalent in most developed countries worldwide. The prevalence of childhood obesity has increased faster than adult obesity in many countries since 1980, with more than 70 countries reporting a doubling of obesity rates. In 2015, there were 107.7 million obese children worldwide (1), and approximately 4 million deaths were attributed to high Body Mass Index (BMI), with 70% of these deaths resulting from cardiovascular disease related to high BMI (2). The prevalence of obesity in children was 5%, while the combined prevalence of obesity and overweight was as high as 23% (2). Obese children have a higher risk of cardiometabolic disease compared to their normal weight peers (3–6), and severe obesity is associated with a higher risk of premature death (7).

Pediatric endocrinology identifies sexual precocity as a prevalent endocrine disorder, as indicated by epidemiological surveys that demonstrate a notable and rapid rise in the incidence of precocious puberty among children (8). Epidemiological studies conducted in Denmark have demonstrated a substantial rise in the prevalence of precocious puberty among girls of Danish origin, up to six-fold, and boys of Danish origin, up to fifteen-fold (9). Similarly, a significant increase in the incidence and prevalence of precocious puberty was observed in both genders during an epidemiological survey in Korea from 2008 to 2014 (10). Precocious puberty can pose a psychological burden on affected children, leading to fear, anxiety, low self-esteem and other psychological disorders (11). Furthermore, it increases the risk of developing hypertension, diabetes and infertility in adulthood (8).

Childhood obesity arises from a complex interplay of various environmental and genetic factors (12). It is primarily caused by an energy imbalance, where caloric intake exceeds energy expenditure, leading to an accumulation of excess body weight and adipose tissue (1). Sedentary behavior, insufficient sleep, and consumption of calorie-dense, nutrient-poor diets are common behavioral causes of obesity (13). A meta-analysis revealed that obesity in girls is a risk factor for early onset of puberty (14). Numerous studies have demonstrated a positive association between BMI and early onset of puberty in girls (15, 16). However, it remains unclear whether precocious puberty is a risk factor for obesity in both boys and girls. This novel question lacks a definitive answer, which motivated our systematic review of population-based studies to evaluate the association between precocious puberty and obesity, general obesity, central obesity, and overweight.

## 2. Materials and methods

### 2.1. Methods

The present meta-analysis adhered to the revised Preferred Reporting Items for Systematic Reviews and Meta-analyses (PRISMA 2020) guidelines (17) and was prospectively registered with the International Prospective Register of Systematic Reviews (PROSPERO) platform with an approval number of CRD42023404479.

### 2.2. Data sources and searches

A comprehensive systematic search was conducted in major electronic databases including PubMed, Cochrane Library, Embase, and Web of Science up to 1 March 2023, without any restrictions on language, country, or study type. The search strategy utilized both medical subject headings (MeSH) and keywords. The search terms used were “Puberty precocious”, “Pubertas Praecox”, “Obesity”, “Overweight” and their variants. In addition, references of the included studies and other published systematic reviews were thoroughly reviewed to identify any additional relevant studies. The complete search strategy is presented in Supplementary Table S1.

### 2.3. Eligibility criteria

The present study aimed to include eligible studies based on pre-defined criteria, which were (1) case-control study, cohort study or cross-sectional study, (2) investigations of the association between precocious puberty and the risk of obesity, central obesity or overweight, (3) reported risk of obesity as the outcome, presented as an adjusted odds ratio (OR) with its corresponding 95% confidence interval (CI). Obesity was considered the primary outcome, and general obesity, central obesity, or overweight were regarded as secondary outcomes. In cases where multiple studies reported data based on the same population, the study with the longest follow-up or the largest number of individuals was included.

Exclusion criteria were conference abstracts, study protocols, duplicate publications, and studies that had no outcomes of interest.

### 2.4. Study selection

According to the pre-defined eligibility criteria, two reviewers (Yibu Kong and Xiaofei Xie) were independently selected to screen the literature for the study. Initial screening was performed based on the title and abstract of the articles, and review articles, duplicate publications, animal experiments, and irrelevant articles were excluded. Subsequently, the full-text of the remaining articles was downloaded and carefully read to identify all the studies that fulfilled the criteria. In case of any discrepancy, the reviewers consulted with Professor Yongji Wang.

### 2.5. Data extraction

The process of extracting data was carried out independently by two reviewers (Yibu Kong and Xiaofei Xie) using a pre-designed table. The table was used to extract relevant information such as the first author, year of publication, country or region where the study was conducted, type of study, sample size, study duration, age of participants, gender of population, diagnostic criteria for obesity, type of obesity and confounding variables adjusted for in the analysis. For the meta analytic calculations, adjusted OR at 95% CI for precocious puberty compared to normal development were extracted. When there are multiple subgroups in the same study, each subgroup is analysed as a separate variable. In case of any disagreement, a third reviewer (Yongji Wang) was consulted to reach a consensus.

### 2.6. Risk of bias assessment

Two reviewers (Yongfu Song and Yongji Wang) independently assessed the methodological quality and level of evidence of the included studies and resolved differences through discussion. The quality of the included studies was assessed using the Newcastle-Ottawa Scale (NOS) for cohort and case-control studies (18). The NOS evaluates the quality of a study based on three main criteria: study population selection, comparability of groups, and outcome or exposure assessment. The total score ranges from 0 to 9 stars. Based on the total score, the study was categorized as low quality (0–3 stars), moderate quality (4–6 stars), and high quality (7–9 stars).

For cross-sectional studies, the American Agency for Health Care Quality and Research's (AHRQ) tool was used to assess study quality (19). If the answer was “yes”, we assigned 1 point, if the answer was “unclear” or “no”, we assigned 0 point, and finally a maximum of 11 points could be awarded, with more points indicating higher study quality. Based on the scores, the studies were categorized as low quality (0–3 points), moderate quality (4–7 points), and high quality (8–11 points).

### 2.7. Statistical analysis

The stata software version 14.0 was used to perform the meta-analysis. The adjusted OR and its 95% CI from the included studies were used to calculate the relation between precocious puberty and obesity, general obesity, central obesity or overweight. Heterogeneity was calculated using *I*^2^-values. If *P* > 0.1 and *I*^2 ^≤ 50%, we used a fixed-effects model. If *I*^2^ > 50%, we used a random-effects model. Considering clinical heterogeneity, a random-effects model was adopted by most studies. The sensitivity analysis was performed to verify the robustness of the overall effects. The funnel plot and Egger's regression test was conducted to statistically assess publication bias.

## 3. Results

### 3.1. Search results

**Figure 1:**
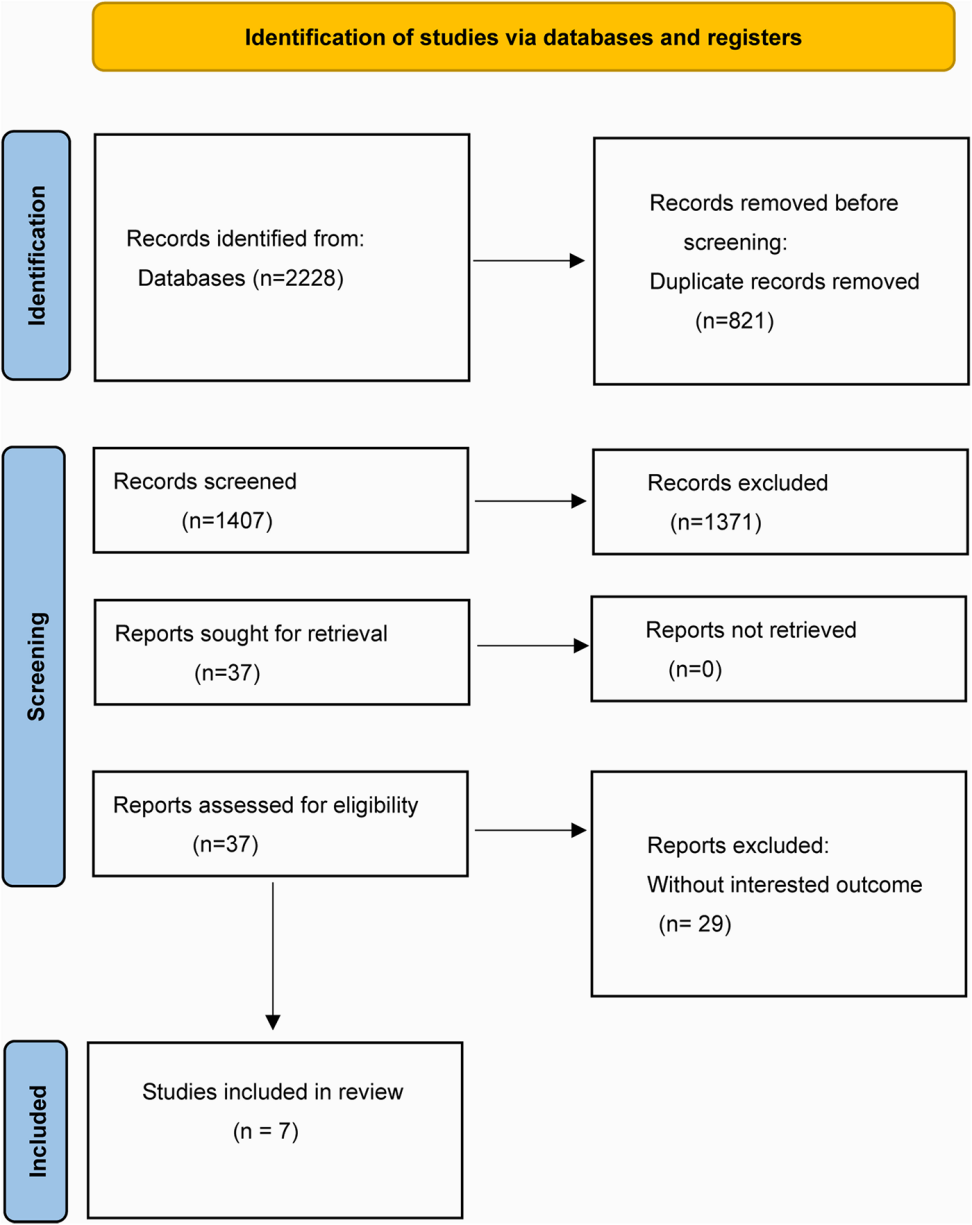
The complete process of eligible literature screening.

A total of 2,228 articles were identified from the systematic search conducted before March 1, 2023, out of which 821 duplicate articles were excluded. Based on the screening of the title and abstract, 1,371 articles were excluded. Finally, seven studies were found to be eligible for inclusion in this meta-analysis (20–26). The process of eligible study selection has been depicted in Figure 1.

### 3.2. Study characteristics

This meta-analysis includes four cohort studies (20, 22, 25, 26), and four cross-sectional studies (21–24), encompassing a total of 65,144 participants (24,464 boys and 40,680 girls) published between 2001 and 2022. One of the included studies contained both cross-sectional and cohort studies (22), and we classified compound obesity as central obesity. Although there were slight variations in the confounding factors such as age, gender, exercise, and energy intake, adjusted estimates were reported in all studies. The characteristics of the seven studies are summarized in Tables 1, 2.

**Table 1:** The characteristics of included studies.

Author	Year	Country	Study types	Sample size	Study period	Age (years)	Confounders adjusted	Scores
Bratberg et al. (20)	2007	Norway	Cohort study	Total: 1,605 Girls: 846; Boys: 697	1992–2001	12–16	Age, exercise, sugar intake, drinking	8
Himes et al. (21)	2004	America	Cross-sectional	Total (girls): 147	2000	8–10	Age, height	6
Majun et al. (22)	2022	China	Cross-sectional*	Total: 44,403 Girls: 22,696; Boys: 21,707	2013–2020	6–18	Age, sex, exercise	7
			Cohort study^Δ^				intake	8
Ribeiro et al. (23)	2006	Portugal	Cross-sectional	Total: 819 Girls: 437; Boys: 382	2001	10–15	School, age	6
Wang (24)	2002	America	Cross-sectional	Total: 3,021 Girls: 1,520; Boys: 1,501	1988–1994	8–14	Age, energy intake, exercise	6
Chen and Wang (25)	2009	America	Cohort study	Total: 411 Girls: 234; Boys: 177	2004–2005	9–15	intake, exercise	7
Adair and Gordon-Larsen (26)	2001	America	Cohort study	Total (girls): 14,738	1994–1996	13–18	Age, parental education, parental obesity, height	7

* indicates a cross-sectional study and ^Δ^ indicates a cohort study.

**Table 2:** The diagnostic criteria for precocious puberty and obesity/overweight.

Author	Year	Diagnosis of precocious puberty	Diagnosis of obesity/overweight	Types of precocious puberty	Types of obesity	Treatment
Bratberg et al. (20)	2007	Boys: Self-reports Girls: Age at menarche Pubertal developmental scale	The International Obesity Task Force Standards (2000)	Early sexual maturation Boys: Pubic hair growth Girls: Earlier menarche	Overweight	Unclear
Himes et al. (21)	2004	Criteria of reynolds and wines and tanner	Center for Disease Control and Prevention growth charts (2000) Overweight: BMI ≥ 85th percentile Obesity: BMI ≥ 95th percentile	Early sexual maturation Thelarche, Pubic hair growth	Obesity Overweight	Unclear
Majun et al. (22)	2022	Cross-sectional: First ejaculation/menarche and the specific time* Cohort study: Tanner stage and physical examination^Δ^	Chinese health industry standards	Early onset of puberty Boys: Ejaculation* Girls: Earlier menarche* Boys: Genitalia development^Δ^ Girls: Thelarche^Δ^	Obesity Central obesity	Unclear
Ribeiro et al. (23)	2006	Tanner stage	The International Obesity Task Force Standards (2000)	Early sexual maturation Thelarche, Genitalia development	Overweight	Unclear
Wang (24)	2002	Tanner stage and the median age	Center for Disease Control and Prevention growth charts (2000) Overweight: BMI ≥ 85th percentile Obesity: BMI ≥ 95th percentile	Early sexual maturation Thelarche, Genitalia development	Obesity Overweight	Unclear
Chen and Wang (25)	2009	Tanner stage and the median age	Center for Disease Control and Prevention growth charts (2000) Overweight: BMI ≥ 85th percentile Obesity: BMI ≥ 95th percentile	Early sexual maturation Thelarche, Genital development	Obesity Overweight	Unclear
Adair and Gordon-Larsen (26)	2001	Unclear	Reference data for obesity: 85th and 95th percentiles of body mass index (1991) Overweight: BMI ≥ 85th percentile Obesity: BMI ≥ 95th percentile	Early sexual maturation	Overweight	Unclear

* indicates a cross-sectional study and ^Δ^ indicates a cohort study.

### 3.3. Risk of bias assessment

According to NOS and AHRQ criteria, the scores of all included cohort and cross-sectional studies are shown in Tables 1, 2. Base on NOS criteria, two studies (20, 22) were scored as 8 (high quality) and two studies (25, 26) were scored as 7 (high quality), and the average score for each study was 7.5, indicating that all cohort studies were of high quality. Base on AHRQ criteria, a study (22) was scored as 7 (moderate quality) and 3 studies (21, 23, 24) were scored as 6 (moderate quality), and the average score for each study was 6.25, representing that all cross-sectional studies were of moderate quality.

### 3.4. Precocious puberty and risk of obesity

**Figure 2:**
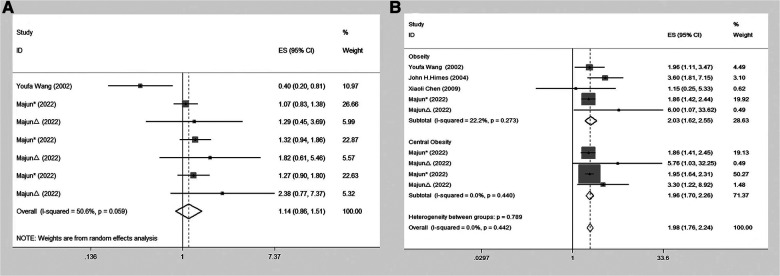
Forest plot of precocious puberty and the risk of obesity. (**A**) In boys; (**B**) in girls.

The present meta-analysis investigated the association between precocious puberty and the risk of obesity, separately for boys and girls. A total of four studies (21, 22, 24, 25) reported on the association between precocious puberty in girls and the risk of obesity. The pooled analysis demonstrated a significant positive association between precocious puberty in girls and an increased risk of obesity (OR = 1.98; 95% CI: 1.76–2.24; *I*^2 ^= 0.0%, *P* < 0.001; Figure 2B). Two studies (22, 24) investigated the association between precocious puberty in boys and the risk of obesity, and the pooled analysis revealed that precocious puberty in boys was not significantly associated with an increased risk of obesity (OR = 1.14; 95% CI: 0.86–1.51; *I*^2 ^= 50.6%, *P *= 0.369; Figure 2A).

### 3.5. Precocious puberty in girls and risk of obesity

**Figure 3:**
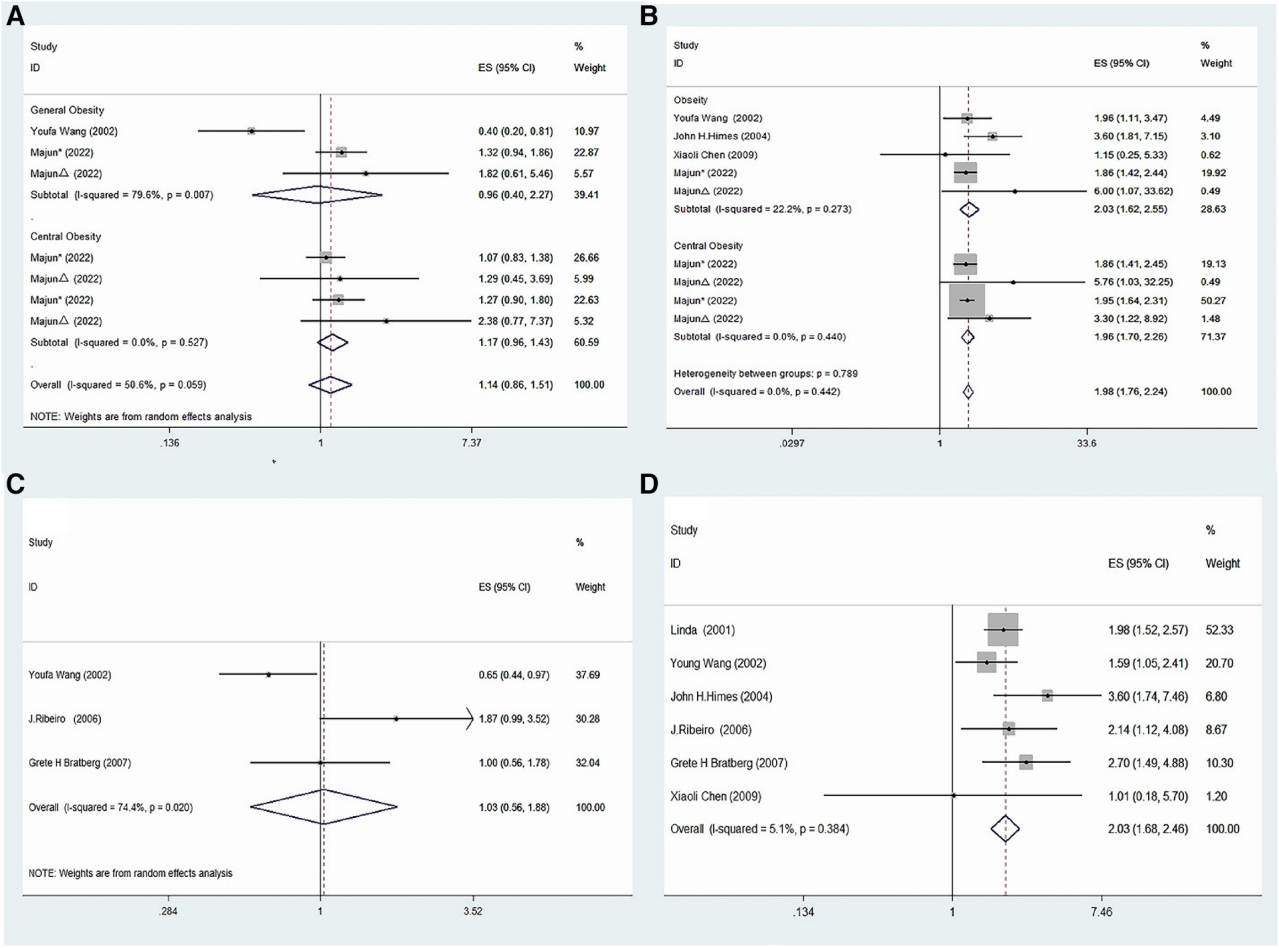
Meta-analysis of specific subgroups. (**A**) Precocious puberty in boys and the risk of various forms of obesity. (**B**) Precocious puberty in boys and the risk of various forms of obesity. (**C**) Precocious puberty in boys and the risk of overweight. (**D**) Precocious puberty in girls and the risk of overweight.

Four studies (21, 22, 24, 25) were included to investigate the association between precocious puberty in girls and the risk of general obesity. The pooled analysis indicated that precocious puberty in girls was significantly associated with a higher risk of general obesity (OR = 2.03; 95% CI: 1.62–2.55; *I*^2 ^= 22.2%, *P *< 0.001; Figure 3B). Only one included study (22) assessed the relationship between precocious puberty in girls and the risk of central obesity, and it demonstrated that precocious puberty in girls was associated with an increased risk of central obesity (OR = 1.96; 95% CI: 1.70–2.26; *I*^2 ^= 0.00%, *P *< 0.001; Figure 3B).

### 3.6. Precocious puberty in boys and risk of obesity

Two studies (22, 24) were included to evaluate the relationship between precocious puberty in boys and the risk of general obesity, and the analysis indicated that there was no significant increase in the risk of general obesity for boys with a history of precocious puberty (OR = 0.96; 95% CI: 0.40–2.27; *I*^2 ^= 79.6%, *P *= 0.922; Figure 3A). Furthermore, one study (22) was included to assess the association between precocious puberty in boys and the risk of central obesity, which indicated that the boys with a history of precocious puberty did not have an increased risk of central obesity (OR = 1.17; 95% CI: 0.96–1.43; *I*^2 ^= 0.0%, *P *= 0.125; Figure 3A).

### 3.7. Precocious puberty in girls and risk of overweight

Six included studies (20, 21, 23–26) assessed the relation between precocious puberty in girls and the risk of overweight and found that precocious puberty in girls had a higher risk of overweight (OR = 2.03; 95% CI: 1.68–2.46; *I*^2 ^= 5.1%, *P *< 0.001; Figure 3D).

### 3.8. Precocious puberty in boys and risk of overweight

Three studies (20, 23, 24) were included in this meta-analysis, which evaluated the association between precocious puberty in boys and the risk of overweight. The pooled analysis revealed that the history of precocious puberty in boys was not associated with an increased risk of overweight (OR = 1.03; 95% CI: 0.56–1.88; *I*^2 ^= 74.4%, *P *= 0.930; Figure 3C).

### 3.9. Publication bias

**Figure 4:**
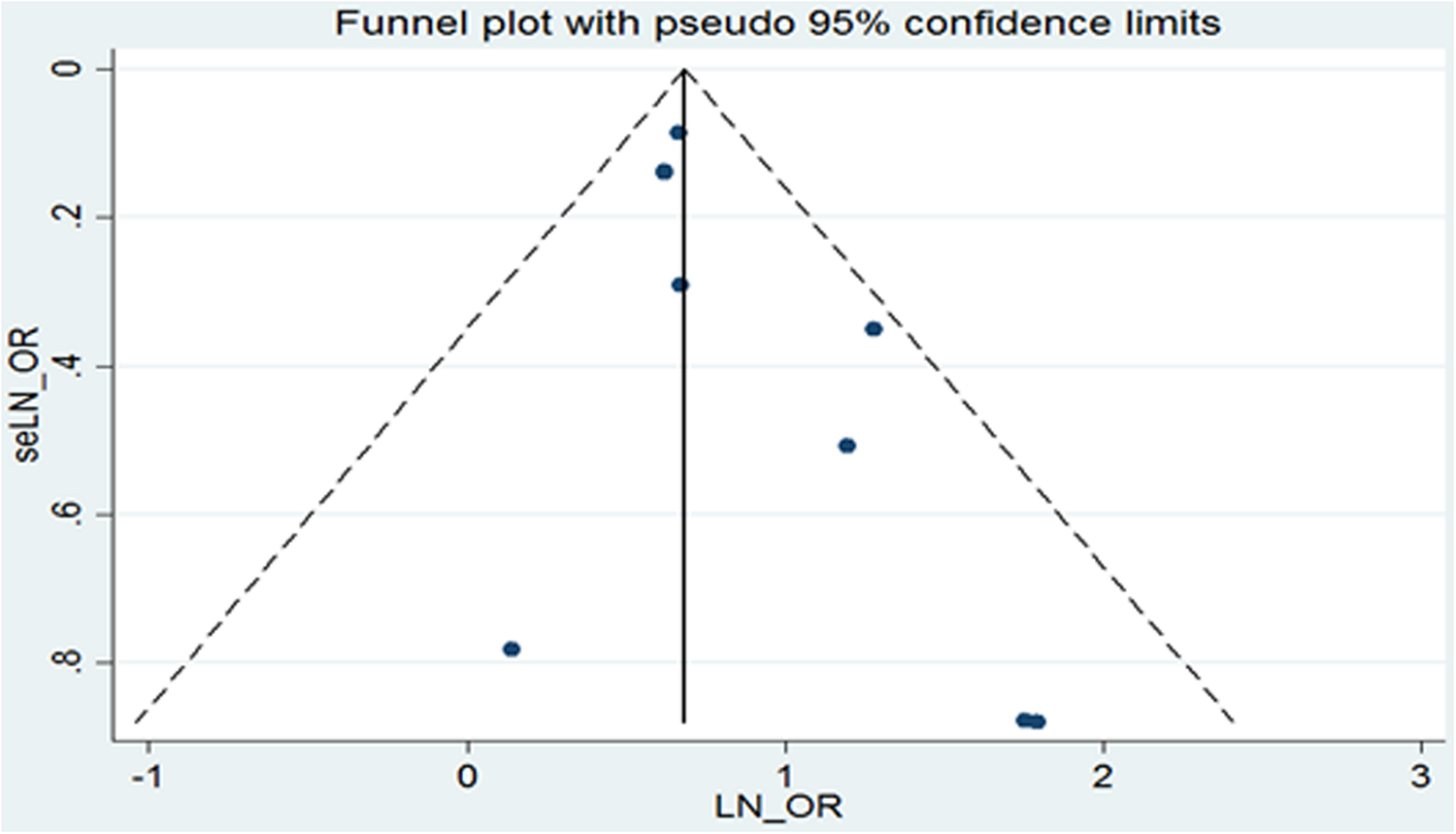
Funnel plot of publication bias.

A visual inspection of the funnel plot showed no evidence of a significant publication bias in the outcome of precocious puberty in girls and the risk of obesity (Figure 4). Egger's regression test (*P* = 0.087) likewise indicated no publication bias in our meta-analysis.

## 4. Discussion

### 4.1. Main findings

In this meta-analysis of 65,144 individuals, including 24,464 boys and 40,680 girls, we investigated the association between precocious puberty and the risk of obesity in girls. Our findings suggest a significant increase in the risk of general obesity, central obesity, or overweight among individuals with precocious puberty in girls. The overall risk increased by 2.03-fold, 1.96-fold, and 2.03-fold for general obesity, central obesity, and overweight, respectively, compared with non-precocious puberty controls. These results suggest that precocious puberty in girls is an independent risk factor for obesity. In contrast, our results show that precocious puberty in boys did not increase the risk of general obesity or overweight. We found a 0.96-fold, 1.14-fold, and 1.01-fold incidence of general obesity, central obesity, or overweight, respectively, among individuals with precocious puberty in boys. These findings suggest that precocious puberty in boys is not a risk factor for general obesity or overweight.

### 4.2. Interpretation of findings

A systematic review and meta-analysis (27) investigated the association between precocious puberty and obesity. The findings showed that girls with precocious puberty had an increased risk of obesity. However, the relationship between precocious puberty and specific types of obesity was not consistent. Some studies reported no significant association between precocious puberty and obesity in boys, while others showed a positive association. Several cross-sectional studies suggested a strong association between precocious puberty and overweight in girls, but limited research has been conducted in boys (24, 26). Our current analysis included more recent and relevant studies and we performed a separate analysis for general obesity, central obesity, and overweight, providing robust evidence regarding the association between precocious puberty and the risk of obesity.

To date, few studies have explored the underlying pathophysiological mechanism linking precocious puberty and obesity. Previous studies have suggested that precocious puberty is associated with early activation of the hypothalamic-pituitary-gonadal (HPG) axis, and identified mutations in the kisspeptin system, AMPK/SIRT signaling, mTOR signaling, and hypothalamic ceramide in precocious puberty (28, 29). Females have a higher number of kiss1 neurons in the anterior ventral periventricular nucleus, which is essential in establishing positive feedback between ovarian steroids and gonadotropin-releasing hormone surge generators and makes females more sensitive to certain metabolic signals than males (29–31). One study suggested that early menarche in girls may lead to obesity (32), possibly due to excessive levels of sex steroids caused by precocious puberty (33). Obesity is associated with leptin, which is directly proportional to body fat stores and acts on certain hypothalamic neurons (30). Leptin can initiate precocious puberty by causing a massive release of nocturnal gonadotropins (34), but excess leptin has been found to inhibit male reproductive function (35).

In the present study, we conducted a subgroup analysis to investigate the association between precocious puberty and obesity, and our results indicate that girls with precocious puberty have a significantly higher risk of general obesity, central obesity or overweight than boys with precocious puberty. Our findings confirm the results of previous reports suggesting that precocious puberty is associated with an increased risk of obesity in girls, while the relationship between precocious puberty and obesity in boys remains inconclusive according to previous studies (25, 36, 37). Previous studies have also shown that boys with precocious puberty are associated with a higher BMI, although the BMI threshold for puberty development in boys is higher than that in girls (38). During pubertal development, significant sex differences exist between obese girls and boys, with increased peripheral conversion of low-potency androgens to estrogens by adipose tissue-aromatase and increased insulin resistance being two potential contributing factors (39). These findings provide further evidence for sex differences in the relationship between precocious puberty and obesity.

In the analyzed studies, we observed significant heterogeneity in the results, which could be attributed to various factors. Firstly, small sample sizes in three studies may have influenced the precision of the results (21, 23, 25), thus requiring larger sample sizes to confirm the relationship between precocious puberty and obesity. Secondly, differences in diagnostic criteria for obesity in the included studies, such as the use of BMI or Waist To Height Ratio (WHTR), as well as varying methods of examination or questionnaire, may have led to discrepancies in the results. Additionally, the diagnosis of obesity was mainly based on electronic health records in some included studies, which may have further contributed to variability in the findings. Thirdly, the geographic distribution of the studies conducted in America, Europe, and Asia may have introduced regional bias, which may affect the generalizability of the results.

### 4.3. Implications and limitations

This meta-analysis aimed to investigate the association between precocious puberty and the risk of obesity. The results suggest that precocious puberty in girls should be considered an independent risk factor for obesity. This finding highlights the importance of identifying high-risk groups of obesity in girls with precocious puberty. However, this meta-analysis also found that the history of precocious puberty in boys does not increase the risk of general obesity, central obesity, or overweight. This suggests that precocious puberty in boys is not a risk factor for these types of obesity.

There are several limitations to this meta-analysis that should be considered. Firstly, only seven studies were included, which may limit the ability to perform subgroup analyses for other types of obesity. Secondly, although the included studies adjusted for confounders, the lack of covariate analyses in this meta-analysis should be acknowledged. Thirdly, the sample size of overweight boys included in the study was small, and larger sample sizes are needed to determine the relationship between early maturity and overweight in boys (20, 23, 24). Fourthly, the diagnostic criteria for obesity varied among the included studies, which may have influenced the final results. Specifically, BMI has been found to be positively correlated with precocious puberty, and as a result, children with precocious puberty may be misclassified as obese or overweight (21). This issue has been previously addressed by the World Health Organization (WHO) Expert Committee on Use of Anthropometrics (40), which provided corrections and evaluated them (41). However, the misclassification of adolescents as obese due to adult-related BMI classification errors can have psychological effects on children (42). Therefore, it is crucial for pediatricians to carefully distinguish between pubertal development and obesity and to select appropriate treatment methods.

### 4.4. Conclusion

Our study indicates that girls with a history of precocious puberty are at an increased risk of obesity. In contrast, boys with precocious puberty do not appear to have an increased risk of general obesity, central obesity, or overweight, although this finding should be interpreted with caution. However, the precise pathophysiological mechanisms underlying this association require further investigation through additional studies. The results of our meta-analysis can provide valuable insight into the prevention and treatment strategies for obesity.

## Data Availability

The raw data supporting the conclusions of this article will be made available by the authors, without undue reservation.
