# Corrigendum: Association between precocious puberty and obesity risk in children: a systematic review and meta-analysis

**DOI:** 10.3389/fped.2023.1283833

**Published:** 2023-09-26

**Authors:** Yongfu Song, Yibu Kong, Xiaofei Xie, Yongji Wang, Na Wang

**Affiliations:** Department of Pediatrics, Hospital Affiliated to Changchun Traditional Chinese Medicine University, Changchun, China

**Keywords:** precocious puberty, general obesity, central obesity, overweight, meta-analysis

A Corrigendum on Association between precocious puberty and obesity risk in children: a systematic review and meta-analysis By Song Y, Kong Y, Xie X, Wang Y and Wang N. (2023) Front. Pediatr. 11:1226933. doi: 10.3389/fped.2023.1226933

**Error in Figure**

In the published article, there was an error in Figure 3 as published. Figure 3A is incorrectly illustrated and does not match the results of the paper. The corrected Figure 3 and its caption appear below.

**Figure 3:**
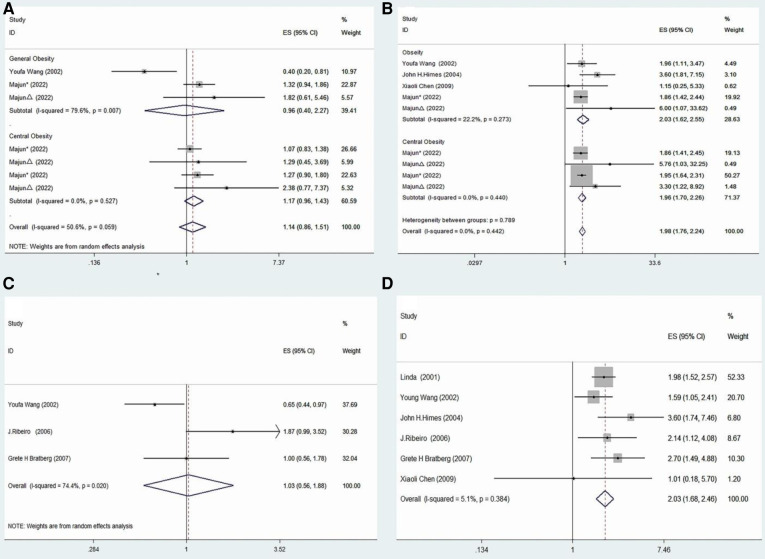
Meta-analysis of specific subgroups. (**A**) Precocious puberty in boys and the risk of various forms of obesity. (**B**) Precocious puberty in boys and the risk of various forms of obesity. (**C**) Precocious puberty in boys and the risk of overweight. (**D**) Precocious puberty in girls and the risk of overweight.

The authors apologize for this error and state that this does not change the scientific conclusions of the article in any way. The original article has been updated.

## Publisher's note

All claims expressed in this article are solely those of the authors and do not necessarily represent those of their affiliated organizations, or those of the publisher, the editors and the reviewers. Any product that may be evaluated in this article, or claim that may be made by its manufacturer, is not guaranteed or endorsed by the publisher.

